# M2-polarized macrophages contribute to neovasculogenesis, leading to relapse of oral cancer following radiation

**DOI:** 10.1038/srep27548

**Published:** 2016-06-08

**Authors:** Makiko Okubo, Mitomu Kioi, Hideyuki Nakashima, Kei Sugiura, Kenji Mitsudo, Ichiro Aoki, Hideki Taniguchi, Iwai Tohnai

**Affiliations:** 1 Department of Oral and Maxillofacial Surgery, Yokohama City University Graduate School of Medicine, Yokohama, Kanagawa 236-0004, Japan; 2 Department of Pathology, Yokohama City University Graduate School of Medicine, Yokohama, Kanagawa 236-0004, Japan; 3 Department of Regenerative Medicine, Yokohama City University Graduate School of Medicine, Yokohama, Kanagawa 236-0004, Japan

## Abstract

Despite the fact that radiation is one of the standard therapies in the treatment of patients with oral cancer, tumours can recur even in the early stages of the disease, negatively impacting prognosis and quality of life. We previously found that CD11b^+^ bone marrow-derived cells (BMDCs) were recruited into human glioblastoma multiforme (GBM), leading to re-organization of the vasculature and tumour regrowth. However, it is not yet known how these cells contribute to tumour vascularization. In the present study, we investigated the role of infiltrating CD11b^+^ myeloid cells in the vascularization and recurrence of oral squamous cell carcinoma (OSCC). In a xenograft mouse model, local irradiation caused vascular damage and hypoxia in the tumour and increased infiltration of CD11b^+^ myeloid cells. These infiltrating cells showed characteristics of M2 macrophages (M2Mφs) and are associated with the promotion of vascularization. M2Mφs promoted tumour progression in recurrence after irradiation compared to non-irradiated tumours. In addition, we found that CD11b^+^ myeloid cells, as well as CD206^+^ M2Mφs, are increased during recurrence after radiotherapy in human OSCC specimens. Our findings may lead to the development of potential clinical biomarkers or treatment targets in irradiated OSCC patients.

Surgery is standard therapy for the treatment of patients with oral cancer, even at advanced stages. However, chemo- and radiotherapy also play major roles in the treatment of advanced OSCC to avoid functional disorders and cosmetic disturbances. Despite advances in treatment modalities, tumours may recur within the irradiated field, leading to a poor prognosis. Thus, improving local control of the primary tumour with radiotherapy would increase the cure rate of oral cancer. To that end, it is necessary to understand how tumour vasculature can be restored after irradiation, given that a local dose should eliminate existing tumour endothelial cells. Since Folkman proposed that tumours cannot grow beyond 2 to 3 mm in size without forming new blood vessels^1^, tumour angiogenesis has been a critical target of cancer therapy; substantial evidence has indicated that vascular endothelial growth factor (VEGF) plays an essential role in developmental angiogenesis. Although anti-angiogenic therapies with anti-VEGF antibodies and other VEGF inhibitors have been popular, their effect is often transitory, and tumours can regrow with increased aggressiveness following cessation of the treatment^2^,^3^. These data indicate that preventing angiogenesis is insufficient to inhibit tumour growth. Tumour vasculature is thought to be dependent on two principal factors. Angiogenesis is derived from the sprouting of endothelial cells from existing tumour vessels or nearby normal vessels^1^, and vasculogenesis is due to colonization of circulating endothelial progenitor cells (EPCs) or BMDCs^4^. Because tumour endothelial cells divide actively and are highly radiosensitive, it is unlikely that any of them could survive the doses given in a typical radiotherapy regimen. Thus, local irradiation may block the angiogenesis pathway, forcing tumour recurrence to rely on the vasculogenesis pathway.

We have previously shown in an intracranial GBM xenograft model that irradiation induces recruitment of BMDCs into tumours, restoring radiation-damaged vasculature by vasculogenesis and thus allowing the growth of surviving tumour cells^5^. Kozin *et al.* also demonstrated in lung and breast tumour models that host-derived BMDC infiltration in tumours was stimulated by local irradiation and facilitates tumour recurrence through paracrine effects on irradiated tumour vasculature^6^. Both studies indicated that CD11b^+^ BMDCs, but not EPCs, make significant contributions to facilitate tumour regrowth after irradiation.

CD11b^+^ myeloid cells recruited into tumours are thought to subsequently differentiate into macrophages. Macrophages surrounding the tumour are referred to as tumour-associated macrophages (TAMs), which are believed to act as key regulators of tumour angiogenesis, migration, metastasis, and treatment resistance^7^. Macrophages are also polarized into two specific phenotypes in response to signals present within individual microenvironments. Pro-inflammatory M1Mφs (classically activated), which are activated by LPS and IFN-gamma, secrete TNF-alpha, IL-12, IL-6 and inducible NO synthase (iNOS) and support T-cell function. In contrast, M2Mφs (alternatively activated), which are activated by IL-4 and IL-13, produce IL-10 and TGF-beta and down-regulate T-cell function. M2Mφs are thought to be anti-inflammatory and immunosuppressive. M1 and M2Mφs also have the ability to suppress and promote tumour progression, respectively^8^. Infiltrated TAMs in tumours are generally characterized as M2 phenotypes and promote tumour growth and vasculature^9^,^10^,^11^. Many clinical studies have also suggested a positive correlation between the number of TAMs and/or M2 profiles in a tumour and increased tumour angiogenesis and metastasis and poor prognosis in cancer patients^12^,^13^,^14^,^15^,^16^. Because monocytes should be polarized into macrophages in peripheral tissues, we hypothesized that CD11b^+^ myeloid cells recruited into irradiated tumours may be differentiated into a proangiogenic phenotype of macrophages. Therefore, our research aimed to investigate whether CD11b^+^ myeloid cells are recruited into oral cancer after irradiation and how they are differentiated into macrophages in relation to tumour relapse, which remains largely uncharacterized.

## Results

### Irradiation causes vascular damage and tumour hypoxia, leading to recruitment of CD11b^+^ myeloid cells into OSCC tumours

**Figure 1:**
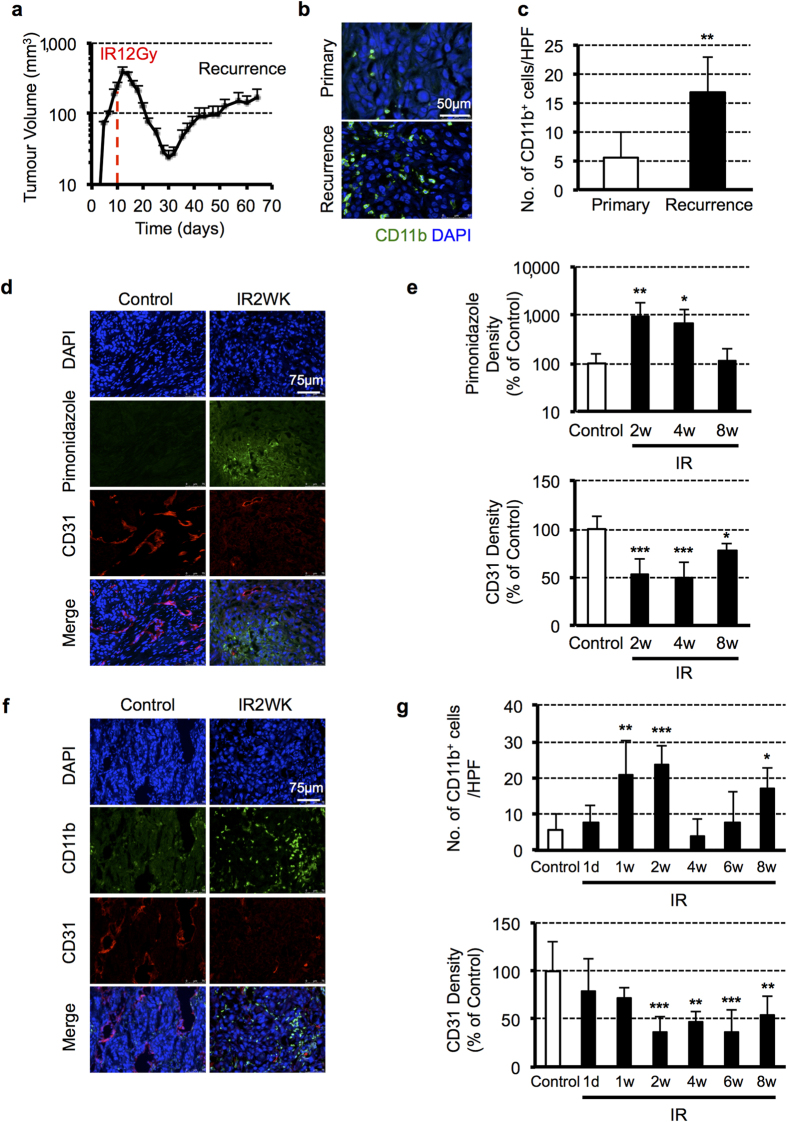
Irradiation causes vascular damage and tumour hypoxia that promotes homing of BM-derived CD11b^+^ myeloid cells into OSCC tumours. (**a**) Growth curve of OSC-19 s.c. tumours treated with 12 Gy local irradiation on day 10. Data shown are means ± SEM, *n* = 4. (**b**) Representative images of IHC for CD11b staining in OSC-19 s.c. tumours before and after irradiation. Scale bars: 50 μm. (**c**) Quantification of CD11b cell influx into the tumour. Data shown are means ± SD. **P < 0.01. (**d**) Representative images of IHC staining for CD31^+^ vascular endothelial cells and pimonidazole^+^ hypoxic areas in OSC-19 tumours two weeks after 12 Gy irradiation. Scale bars: 75 μm. (**e**) Quantification of vessels and hypoxic areas in the tumour following irradiation. Data shown are means ± SD. *P < 0.05; **P < 0.01; ***P < 0.001 versus control. (**f** ) IHC staining for CD11b^+^ cells and CD31^+^ vascular endothelial cells in OSC-19 tumours two weeks after 12 Gy irradiation. Scale bars: 75 μm. (**g**) Quantification of CD11b^+^ cells and vessels in the tumour following irradiation. Data shown are mean ± SD. *P < 0.05; **P < 0.01; ***P < 0.001 versus control. IR, irradiation.

To assess alterations in the tumour microenvironment following radiotherapy, we established an OSCC xenograft mouse model using OSC-19 cells. As shown in Fig. 1a, s.c. implanted tumours grew progressively; however, 12 Gy local irradiation caused the tumours to regress, followed by regrowth in several weeks. Tumour samples following irradiation were collected when the tumours grew back to their pre-irradiation sizes and were analysed by immunofluorescence staining to compare the influx of CD11b^+^ cells with that of non-irradiated control tumours. We found that recurrences contained significantly higher numbers of CD11b^+^ cells (Fig. 1b,c). Similar results were obtained from other OSCC s.c. tumour models using HSC-3 (tongue) and YCU-OR891 (oral floor) cells (Supplementary Fig. S1).

The results of previous reports^5^,^17^,^18^ suggested that CD11b^+^ cell recruitment was induced by hypoxia. To test this, we investigated hypoxia levels in tumours using pimonidazole staining along with blood vessel staining in a time course analysis. We found that hypoxic areas were extended in the tumours, starting from two weeks after irradiation, and CD31^+^ vascular endothelial cells were significantly reduced (Fig. 1d,e). Interestingly, further analysis showed that the highest numbers of CD11b^+^ cells were recruited at the same time point and were inversely proportional to CD31 density (Fig. 1f,g). These data suggest that local tumour irradiation causes vascular damage and hence tumour hypoxia, which may be the stimulus for the recruitment of CD11b^+^ cells into tumours.

### Analysis of BMDC components in OSCC tumour-bearing mice

**Figure 2:**
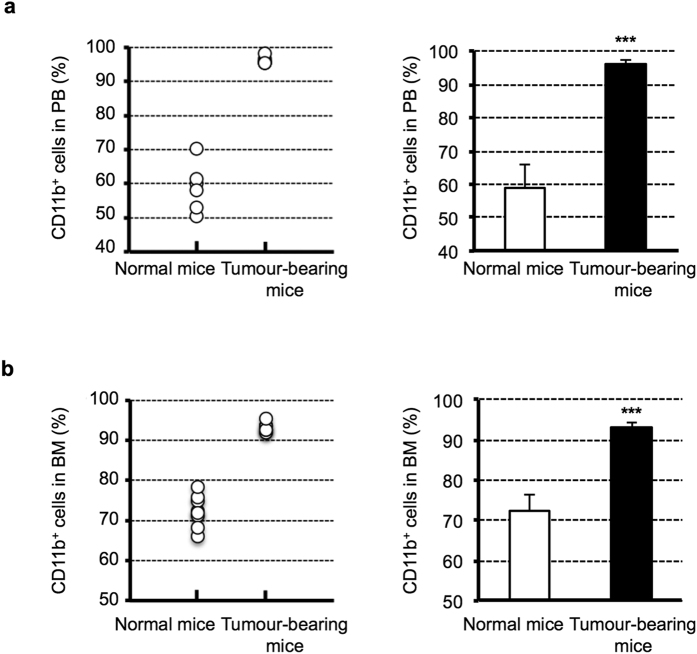
The proportion of CD11b^+^ cells in PB and BM of normal mice and OSC-19 tumour-bearing mice. (**a**) PB was prepared by collecting whole blood from normal mice and OSC-19 tumour-bearing mice and eliminated red blood cells. CD11b positive rate in PB was comparative analysed by FACS. (**b**) BM was prepared from normal mice and OSC-19 tumour-bearing mice as previously described in Methods. CD11b positive rate in BM was comparatively analysed by FACS. Data shown are means ± SD. *n* = 6–8 per group, ***P < 0.001. PB, peripheral blood; BM, bone marrow.

It was previously reported that monocyte precursors were increased in the BM, spleen and peripheral blood (PB) of tumour-bearing mice in various cancers^19^,^20^. Therefore, we first determined the proportion of CD11b^+^ cells in the PB and BM of normal mice and OSC-19 tumour-bearing mice by FACS analysis. In both the PB and the BM, the percentage of CD11b^+^ cells obtained from OSC-19 tumour-bearing mice was significantly higher than that of cells from normal mice (Fig. 2a,b). These data suggest that not only blood cells but also BMDCs may be altered by tumours, most likely by the secretion of cytokines and chemokines.

### CD11b^+^ dominant BMDCs accelerate new blood vessel formation in tumours at the initial stage when tumours were injected

**Figure 3:**
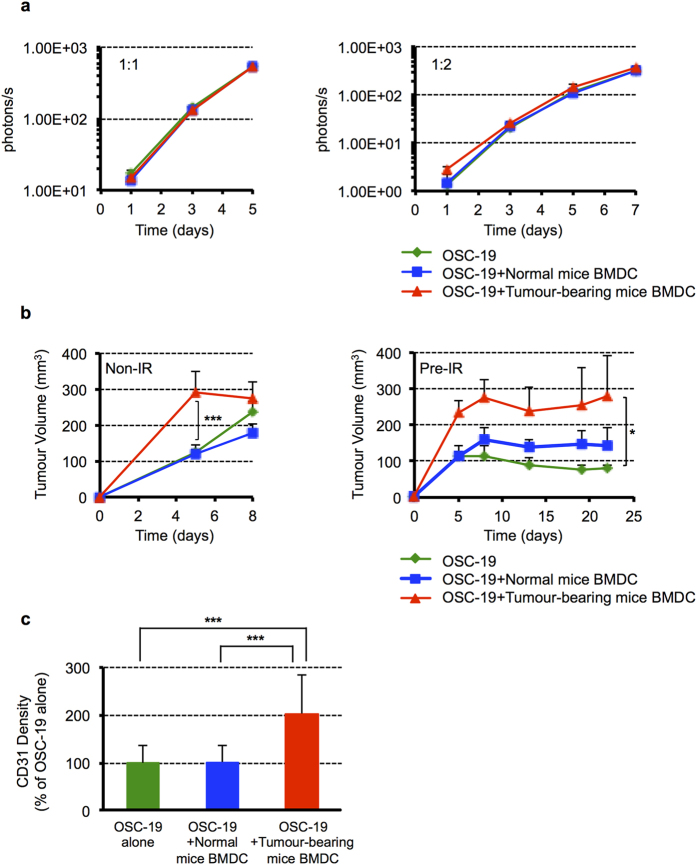
CD11b^+^ dominant BMDCs may stimulate blood vessels formation and promote tumour growth. **(a**) OSC-19-luc cells (4 or 8 × 10^3^ cells) were co-cultured with BMDCs (8 × 10^3^ cells) of normal mice or OSC-19 tumour-bearing mice in 24-well plates in quadruplicate. Bioluminescent signals were determined using the IVIS system on the indicated date. Data shown are means ± SD. (**b**) OSC-19 cells (2 × 10^6^ cells) were co-injected with BMDCs (2 × 10^6^ cells) of normal mice or OSC-19 tumour-bearing mice into non-IR or pre-IR sites of nude mice. Tumour size was measured on the indicated date. Data shown are means ± SEM, *n* = 5–7 per group. *P < 0.05; ***P < 0.001. (**c**) Quantification of CD31 density of IHC in the early stage (day10) of the tumour at non-IR site. Data shown are means ± SD. ***P < 0.001. Non-IR, non-irradiated; Pre-IR, previously-irradiated; BMDC, bone marrow-derived cell.

We then investigated whether BMDCs of OSC-19 tumour-bearing mice with higher numbers of CD11b^+^ cells affected cancer cell biology *in vitro* or tumour growth *in vivo*. To test this, OSC-19-luc cells were co-cultured with BMDCs of normal control mice or tumour-bearing mice, and the proliferation of OSC-19-luc cells was assessed by the IVIS system. The results showed no significant difference between the control and tumour-bearing BMDCs with respect to OSC-19 cell proliferation, suggesting that in spite of different CD11b positive rates, BMDCs have no effect on OSC-19 cell proliferation (Fig. 3a).

We next examined the effect of BMDCs on the growth of OSC-19 s.c. tumours in nude mice. In this experiment, BMDCs derived from normal control mice or tumour-bearing mice were co-injected with OSC-19 cells into non-IR or pre-IR sites, in which irradiation eliminated pre-existing blood vessels, causing the “tumour bed effect”^21^. Tumour nodules were initially formed at both non-IR and pre-IR sites when OSC-19 cells were injected with BMDCs of tumour-bearing mice. At non-IR sites, OSC-19 tumours injected with BMDCs of tumour-bearing mice grew progressively in the early stages of tumour growth. In contrast, the tumours with BMDCs of tumour-bearing mice grew progressively over the period of the experiment at the pre-IR site (Fig. 3b). We next examined blood vessel formation in the early stages of tumour growth in non-IR sites. We observed twice the density of CD31 endothelial cells in tumours when OSC-19 cells were co-injected with BMDCs of tumour-bearing mice as compared to those of normal control mice (Fig. 3c). These results suggest that CD11b^high^ BMDCs derived from tumour-bearing hosts may stimulate new blood vessel formation in the initial stage, when tumours have limited blood supply.

### M2Mφs are polarized from CD11b^+^ myeloid cells in OSC-19 tumours after irradiation

**Figure 4:**
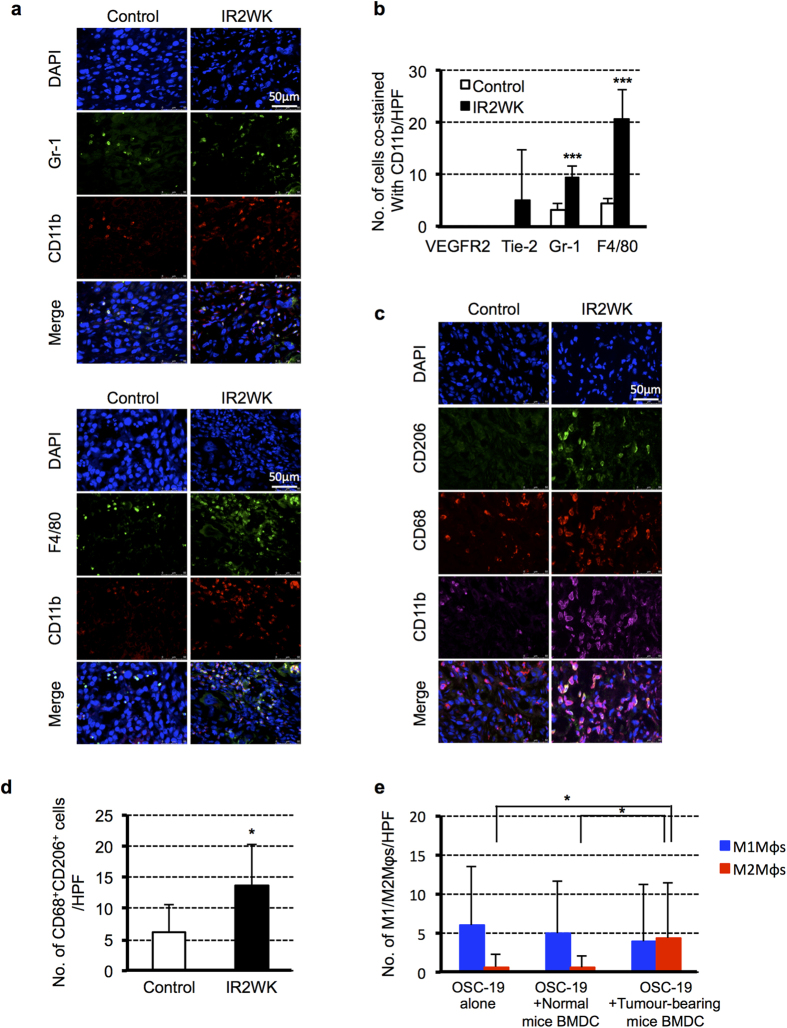
Characterization of CD11b^+^ infiltrating cells into OSC-19 tumours after irradiation. (**a)** Representative images of IHC for DAPI (blue), CD11b (red), and other markers (Gr-1 or F4/80: green) in OSC-19 tumours two weeks after irradiation. Scale bar: 50 μm. (**b**) Quantification of positive staining for VEGFR2, Tie-2, Gr-1, or F4/80 co-stained with CD11b. Data shown are means ± SD. ***P < 0.001. (**c**) Representative images of IHC for CD68^+^CD206^+^ M2Mφs and CD11b^+^ cells in OSC-19 tumours two weeks after irradiation. Scale bar: 50 μm. (**d**) Quantification of M2Mφs localized in peritumoural areas. Data shown are means ± SD. *P < 0.05. (**e**) Quantification of CD68^+^iNOS^+^ M1Mφs and CD68^+^CD206^+^ M2Mφs in the tumours of Fig. 3c. Data shown are means ± SD. *P < 0.05. Mφs, macrophages; BMDC, bone marrow-derived cell.

We next characterized the CD11b^+^ cells recruited into tumours after irradiation. Double immunostaining with CD11b and F4/80, Gr-1, Tie-2 or VEGFR2 revealed that CD11b^+^F4/80^+^ cells were the most common population, followed by CD11b^+^Gr-1^+^ cells. Subsets of CD11b^+^ cells, however small the number, expressed Tie-2 antigen, categorizing them as Tie2-expressing macrophages (TEMs). TEMs have been reported to be highly proangiogenic and to interact with the formation of tumour blood vessels^22^. CD11b^+^VEGFR^+^ circulating precursor cells that contribute to the structural luminal surface of capillaries were not detectable. These results indicate that recruited CD11b^+^ cells are highly related to the macrophage lineage (Fig. 4a,b). We then analysed the characteristics of the macrophage lineage recruited in tumours. We found that CD68^+^CD206^+^ M2Mφs were mainly localized in peritumoural areas and that their number in tumours after irradiation was significantly higher than those in non-irradiated controls (Fig. 4c,d). We next analysed the number of M1 or M2Mφs in tumours, as shown in Fig. 3c. While M2Mφs were significantly induced in OSC-19 tumours co-injected with the BMDCs of tumour-bearing mice compared to that of normal mice, the number of M1Mφs was not significantly affected (Fig. 4e). Furthermore, most of the CD68^+^CD206^+^ M2Mφs co-expressed CD11b antigen (Fig. 4c), indicating that recruited CD11b^+^ cells induced by irradiation are polarized into M2Mφs, resulting in contributions to tumour progression.

### M2Mφs may regulate tumourigenicity in tumour regrowth

**Figure 5:**
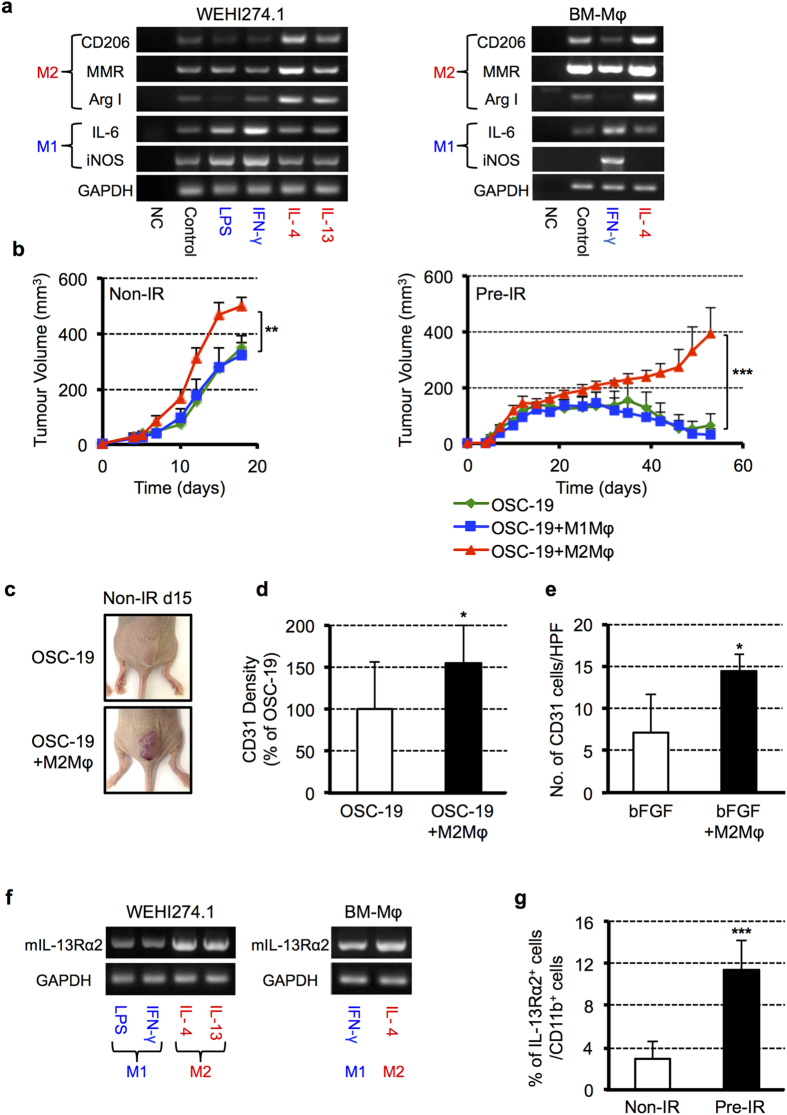
M2Mφs enhance tumourigenicity by accelerating tumour vascularization. (**a**) RT-PCR showed that 24-hours of stimulation with IFN-γ/LPS and IL-4/IL-13 induced the polarization of WEHI274.1 cells and BM-Mφs into M1 and M2Mφs, respectively. (**b**) OSC-19 cells (2 × 10^6^ cells) were co-injected with BM-M1Mφs or BM-M2Mφs (1 × 10^6^ cells) into non-IR or pre-IR sites of nude mice. Tumour size was measured on the indicated date. Data shown are means ± SEM, *n* = 4–5 per group. **P < 0.01, ***P < 0.001. (**c**) The photographs were taken on day 15 after tumour implantation into non-IR site. (**d**) Quantification of CD31 density of IHC in the early stage (day 16) of the tumour at non-IR site. Data shown are means ± SD. *P < 0.05. (**e**) BM-M2Mφs increased the number of CD31 endothelial cells in Matrigel plugs *in vivo.* Data shown are means ± SD, *n* = 4 per group. *P < 0.05. (**f**) RT-PCR showed higher expression of IL-13Rα2 in M2Mφs derived from WEHI274.1 cells and BMDCs rather than M1Mφs. (**g**) FACS analysis showed that IL-13Rα2 expression in CD11b^+^ cells recruited into tumours grown at pre-IR sites was significantly higher than that at non-IR sites. Data shown are means ± SD, *n* = 6 per group. ***P < 0.001. BM-Mφ, bone marrow-derived macrophage.

To examine the function of M1 and M2Mφs in tumour growth and regrowth following irradiation, we first confirmed the induction of macrophage polarization in WEHI274.1 cells and BM-Mφs obtained from nude mice. Cells were incubated with various cytokines to induce differentiation into M1 or M2Mφs. RT-PCR showed that IFN-γ/LPS and IL-4/IL-13 stimulation induced the polarization of M1 and M2Mφs, respectively (Fig. 5a). We next investigated whether BM-M1Mφs and BM-M2Mφs can influence OSC-19 cell proliferation *in vitro*. OSC-19-luc cells were incubated with BM-M1Mφs or BM-M2Mφs, and cell proliferation was assessed by the IVIS system. As shown in Supplementary Fig. S2, there was no significant difference in OSC-19 cell proliferation between the M1 and M2Mφs-co-cultured groups, suggesting that neither M1 nor M2Mφs had an effect on OSC-19 cell proliferation. We next examined the effect of these cells on tumour growth *in vivo*. We injected OSC-19 cells into non-IR or pre-IR sites alone or in the presence of BM-M1Mφs or BM-M2Mφs (Supplementary Fig. S3). Tumour growth with BM-M2Mφs was promoted until day 18 in the non-IR site. Tumour growth at the pre-IR site was significantly accelerated in the presence of BM-M2Mφs over a long period compared to BM-M1Mφs or even tumour cells alone (Fig. 5b). As shown in Fig. 5c, tumours with BM-M2Mφs at non-IR sites appeared haemorrhagic; therefore, we examined blood vessel formation. CD31^+^ vessel cells were significantly increased in tumours when OSC-19 cells were co-injected with BM-M2Mφs compared to controls (Fig. 5d). Furthermore, we investigated the role of BM-M2Mφs in blood vessel formation without cancer cells. As shown in Fig. 5e and Supplementary Fig. S4, BM-M2Mφs promoted tube-like formation in CD31^+^ endothelial cells in a Matrigel plug assay in nude mice compared with controls. These results suggest that BM-M2Mφs enhance tumourigenicity by promoting tumour vasculature, especially when local endothelial cells are eradicated by irradiation.

### The portion of CD11b^+^ myeloid cells and M2Mφs recruited into tumours after irradiation expresses IL-13Rα2

IL-13Rα2, one of the IL-13 receptors, is highly expressed in some cancer cells, including GBM^23^ and OSCC^24^; therefore, targeting this receptor, such as CAR-T and IL13-PE, would be new strategy for these cancer patients^25^. Interestingly, recent reports have shown that macrophages recruited by inflammation in ulcerative colitis and pulmonary fibrosis also express IL-13Rα2^26^,^27^. We therefore investigated whether CD11b^+^ cells and M2Mφs recruited into tumours following irradiation express IL-13Rα2. To test our hypothesis, we examined the expression levels of IL-13Rα2 in M1 and M2Mφs derived from WEHI274.1 cells and BMDCs. RT-PCR showed higher levels of IL-13Rα2 expression in both M2Mφs (Fig. 5f). Furthermore, we compared the IL-13Rα2 expression in CD11b^+^ cells recruited into OSC-19 tumours at non-IR and pre-IR sites by FACS analysis. Interestingly, the percentage of IL-13Rα2^+^ cells in CD11b^+^ cells in tumours grown at pre-IR sites was significantly higher than that of non-IR sites (Fig. 5g). These results suggest that IL-13Rα2 is a potent molecular target for the depletion or ablation of CD11b^+^ cells and for M2Mφs to inhibit reorganization of blood vessels following radiation.

### CD11b^+^ myeloid cells and M2Mφs are increased in recurrences of human OSCC clinical samples

**Figure 6:**
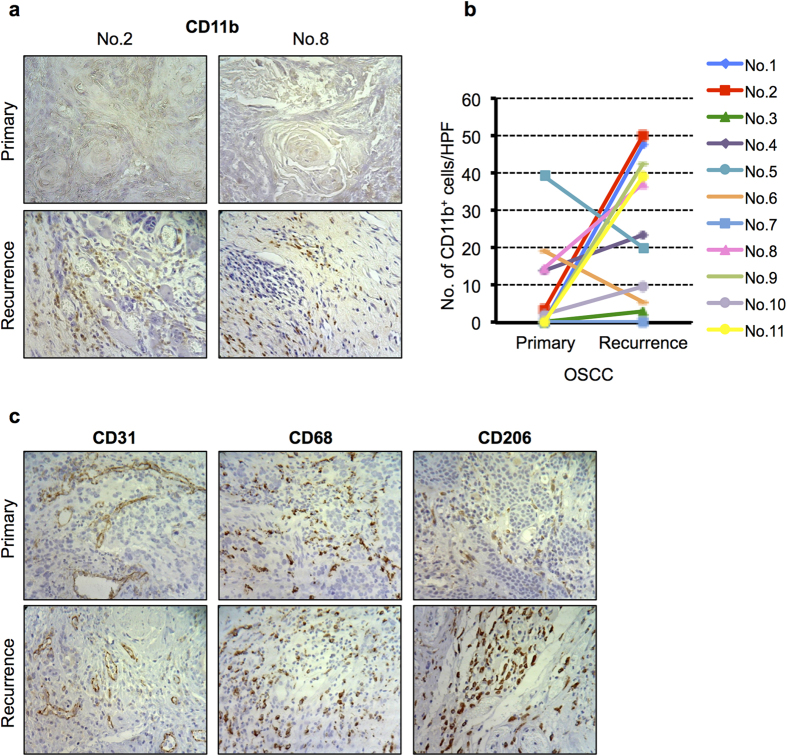
Immunohistochemical analyses of human OSCC clinical specimens. (**a**) Representative staining for CD11b expression in OSCC clinical samples obtained from primary and recurrent tumours. (**b**) Quantification of CD11b based on IHC with CD11b staining. Increased numbers of CD11b^+^ cells in recurrent human OSCCs were seen in eight of eleven samples. *P < 0.05. (**c**) Representative staining for CD31, CD68, and CD206 expression in OSCC clinical samples obtained from primary and recurrent tumours.

To determine the potential clinical relevance for patients with OSCC, we validated our findings from a mouse model in humoral tumour tissue specimens. Immunohistochemical analyses were performed using human OSCC specimens obtained from biopsy samples before treatment and from recurrent tumours after radiotherapy in the same individuals. We found higher levels of CD11b^+^ cells in the recurrent tumours than in the untreated primary tumours in eight out of eleven paired samples (Fig. 6a,b). We then further evaluated the expression of CD31, CD68, and CD206 in the same specimens and found that CD206^+^ M2Mφs was also increased in recurrences, while the recurrent tumours showed lower levels of CD31 vasculature (Fig. 6c).

## Discussion

The present study demonstrated that local irradiation promotes the influx of CD11b^+^ myeloid cells into OSCC, followed by polarization towards M2Mφs, contributing to re-organization of the vascular network in the tumour. Data presented herein show for the first time that irradiation induces CD68^+^CD206^+^ M2Mφs polarization that promotes neovascularization in OSCC in a mouse model.

We have shown that irradiation induces recruitment of CD11b^+^ BMDCs into OSCC. This is consistent with previous studies showing that an increased influx of CD11b^+^ cells was found in irradiated tumours in several cancer models^6^,^15^,^28^. We also previously reported in GBM specimens that there was a significant increase in CD11b^+^ cell infiltration in recurrent tumours compared with their matched primary tumour specimens, which is consistent with our current OSCC data. The stimulus for this phenomenon is that increased tumour hypoxia occurred due to the loss of blood vessels. Hypoxia-inducible factor-1α (HIF-1α) is a crucial factor in the induction of BMDC influx. Various animal studies showed that HIF-1α regulates many chemokines that may induce recruitment of BMDCs into tumours, including chemokine ligand 2 (CCL2 or MCP1)^29^, colony-stimulating factor-1 (CSF-1)^30^,^31^,^32^, and stromal-derived factor-1 (SDF-1)^5^,^17^. The present study also demonstrates that hypoxia is prominently increased in OSC-19 tumours as it initiates recruitment of CD11b^+^ cells into the tumour.

Previous studies reported that CD11b^+^ monocyte precursors were increased in the BM, spleen, and PB of tumour-bearing mice in colon adenocarcinoma, mammary adenocarcinoma, and lymphoma. Moreover, those immature myeloid cells are also further produced in the PB of patients with cancer^19^,^20^. The increases in these cells in the PB are thought to be due to release from the BM by cytokines and chemokines secreted by tumour cells. We also found a higher proportion of CD11b^+^ cells in the PB and BM of OSC-19 tumour-bearing mice, suggesting that BMDCs can be altered by tumours, likely secretory cytokines and chemokines.

BMDC accumulation in OSC-19 tumours after local irradiation was largely composed of CD11b^+^ myeloid cells. Subclass analyses of these cells showed high co-expression of F4/80, indicating that they are of the macrophage lineage. TEMs, which have shown the proangiogenic activity of myeloid cells in tumours, were small in number, which is consistent with a previous report^22^. De Palma *et al.* demonstrated in mouse xenografts that conditional depletion of TEMs prevented angiogenesis and delayed tumour growth. We also reported that approximately half of the CD11b^+^ myeloid cells recruited into the irradiated GBM were TEMs^5^. Inhibiting TEM recruitment into tumours by pharmacologically interfering with the CXCL12/CXCR4 axis increased the efficacy of radiation or vascular-disrupting agents in GBM and murine mammary tumours^5^,^33^. However, inhibition of CXCL12 by AMD3100 had no effect on the regrowth of OSC-19 tumours after irradiation (data not shown), indicating that the proportion of TEMs and chemokine for recruitment is likely different from the types of histology, advancement, or location of the tumours. CD11b^+^Gr-1^+^myeloid cells, known as myeloid-derived suppressor cells (MDSCs), that have been reported accumulate in several tumours, suppress tumour immunity and thus promote tumour growth^34^. In our study, the amount of MDSC accumulation in tumours was significantly increased after irradiation, but not as much as in CD11b^+^F4/80^+^ cells. Moreover, Ahn *et al.* suggested that CD11b-neutralizing antibodies enhanced tumour responsiveness to radiation in transplanted tumours, whereas depletion of Gr-1^+^ cells did not show the same effect. Although the possible involvement of CD11b^+^Gr-1^-^Ly-6C^hi^ monocytic MDSCs in tumour relapse after radiation may not be excluded, CD11b^+^ monocytic cells, but not CD11b^+^ neutrophils or granulocytes, seem to be more critical as a proangiogenic population^28^.

M2Mφs have been reported to have protumoural functions, including functions in angiogenesis and alterations in invasiveness. In our study, M2Mφs had no effect on OSC-19 cell proliferation *in vitro*, while they promoted tumour growth and regrowth *in vivo*. This effect was more significant and lasted much longer in tumours injected at pre-IR sites. CD31 endothelial cells in the tumours of non-IR sites showed that OSC-19 tumours injected with M2Mφs formed more blood vessels. These results suggested that the effect of M2Mφs on neovascularization acts in more ischaemic conditions and contributes to relapse in OSCC tumours. Indeed, in conditions such as wound and ischaemic heart disease models, polarization of the M2 phenotype is promoted in infiltrating macrophages. Oxygen deprivation functionally links to progressive induction of proangiogenic M2Mφs^35^.

We have previously reported in a GBM model that tumour vascularization mainly relies on angiogenesis in normal conditions; however, when local irradiation inhibits angiogenesis and induces hypoxia, vasculogenesis predominates. In this study, CD31 endothelial cells decreased to 36% of control levels by 2 weeks after 12 Gy irradiation so that angiogenesis was inhibited by irradiation in an OSCC model. Because BMDCs of tumour-bearing mice also promoted tumour growth in non-IR sites, it is possible that angiogenesis was also stimulated. However, the effect of tumour-bearing BMDCs was limited to the initial stage of non-IR tumours and was more significant in pre-IR sites; therefore, BMDCs more likely contribute to vasculogenesis in irradiated tumours. Proangiogenic M2Mφs are reported to secrete several angiogenic factors, such as VEGF, IL-8 (CXCL8), bFGF (basic fibroblast growth factor), TP (thymidine phosphorylase), and MMP (matrix metalloproteinase)^11^,^36^, recolonizing and stabilizing tumour vasculature after irradiation, thereby supporting any remaining viable tumour cells. However, the specific mechanism underlying this process is not well understood, and further studies will be necessary.

The observation of increased CD11b^+^ myeloid cells in recurrence also has clinical implications. Evidence has accumulated suggesting that recruitment of myeloid cells into tumours is correlated with clinical outcomes accompanied by treatment. A previous study reported that macrophage density positively correlated with microvessel counts and negatively correlated with patient relapse-free survival in lung cancer^37^. Balermpas *et al.* also reported that CD163^+^ macrophage expression predicts an unfavourable clinical outcome in HNSCC after definitive chemoradiotherapy^15^. In the present study, CD206^+^ M2Mφs was also increased in recurrences of OSCC specimens after radiotherapy. These results suggest that increased myeloid cells or TAMs in tumours or PB may be independent prognostic markers. Local and distant recurrence occurs in approximately 30% of OSCC. Hence, it is crucial to identify novel biomarkers that predict patients at high risk of relapse.

Although several TAM-targeting cancer therapies have been explored, whole TAM depletion may also affect monocyte/macrophage-mediated host defence. Therefore, a M2Mφs-specific targeting agent would need to exert high anti-tumour effects with radiation while minimizing toxicity^38^. To our knowledge, there are very few methods of specific molecular targeting for M2Mφs. Cieslewicz *et al.* developed a unique peptide called M2pep that preferentially binds to M2Mφs, including TAMs^39^. In this study, we found that M2Mφs polarized after radiation highly expressed IL-13Rα2; therefore, this molecule would be a promising target as a new strategy combined sequentially with radiotherapy. It has been demonstrated that IL13-PE composed of IL-13 and truncated *Pseudomonas* exotoxin (PE) directly killed IL-13Rα2 positive cells^40^. Therefore, IL13-PE has the potential to generate synergistic antitumour effects, directly killing tumour cells and specifically ablating protumoural M2Mφs.

In summary, our data suggest that local irradiation induces recruitment of CD11b^+^ myeloid cells into OSCC and that these are polarized towards M2Mφs shortly before tumours start to regrow. M2Mφs rather than CD11^+^ myeloid cells may contribute to neovasculogenesis and relapse in OSCC after radiation.

## Methods

### Cell lines

OSC-19 and HSC-3 cell lines (human squamous cell carcinoma of the tongue) were purchased from the Japanese Collection of Research Bioresources. YCU-OR891 cells (human squamous cell carcinoma of the oral floor) were obtained from Dr. Oridate of Yokohama City University. OSC-19-luc cells were retrovirally transduced with the luciferase gene, as previously described^5^. The WEHI274.1 cell line (murine monocyte) was obtained from Dr. JM Brown (Stanford University). Cells were maintained in PRMI1640 (YCU-OR891) or DMEM (all other cells) containing 10% foetal bovine serum (FBS). All subsequent experiments were approved by the Ethics Committee of Yokohama City University (registration number B100513003).

### Animals

Female 4–7-week-old BALB/c nude mice weighting 14–18 g were purchased from SLC Japan. All studies were conducted in accordance with protocols approved by the Institutional Animal Care and Use Committee at Yokohama City University.

### Isolation of BMDCs

BMDCs were harvested by flushing the femurs and tibias of female BALB/c nude mice with RPMI1640 Glutamax (Thermo Fisher Scientific) supplemented with 10% FBS using a 25G needle and a syringe. BMDCs were passed through a 70 μm cell strainer, and red blood cells were removed by NH_4_Cl solution. BMDCs of OSC-19 tumour-bearing mice were obtained from female BALB/c nude mice previously implanted with OSC-19 cells.

### Cytokine-stimulated macrophage phenotypic polarization

For BMDC differentiation into BM-derived macrophages (BM-Mφs), BMDCs were cultured for an additional 7 days in the same medium supplement with 10 ng/ml recombinant macrophage colony-stimulating factor (M-CSF) (Peprotech). The efficiency of differentiation was confirmed by FACS analysis for CD11b and F4/80 antigen surface expression (Supplementary Fig. S5). To induce the macrophage phenotypic polarization, WEHI274.1 cells and BM-Mφs were treated for 24 hours with 100 ng/ml IFN-γ (Peprotech) or 200 ng/ml LPS (SIGMA) for M1Mφs and 20 ng/ml IL-4 (R&D systems) or 20 ng/ml IL-13 (Miltenyi Biotec) for M2Mφs.

### Reverse transcription-PCR

Total RNA was isolated using RNeasy, and reverse transcription was performed using SuperScript^TM^III Reverse Transcriptase (Thermo Fisher Scientific). PCR with TaKaRa Ex Taq (Takara) was performed with primers (Sigma Genosys), as shown in Supplementary Table S1^41^,^42^,^43^,^44^,^45^. PCR products were viewed using Dolphin-View DV-18 (Kurabou).

### Co-culture study

For two co-culture studies, OSC-19-luc cells were plated in 24-well plates with or without BMDCs of normal control mice or OSC-19 tumour-bearing mice and with or without BM-derived M1Mφs (BM-M1Mφs) or BM-derived M2Mφs (BM-M2Mφs) at indicated ratios. Then, proliferation of OSC-19-luc cells was assessed via the enzymatic activities of luciferase using the IVIS system (IVIS Lumina II ver.4, Caliper) on the indicated date.

### Xenograft tumour mouse model and irradiation

OSC-19 cells (3 × 10^6^ cells) were subcutaneously (s.c.) implanted on the back of a female BALB/c nude mouse. Tumour volume (V) was calculated with callipers as V = L × W^2^ × 0.5 (L, length; W, width). When the tumour volume reached approximately 200 mm^3^, 12 Gy of local irradiation was administered (HITACH X-ray MBR-1520-4) using a specialized lead jig. For the pre-IR model, 12 Gy of irradiation was performed before tumour implantation. For the two co-injection studies, OSC-19 cells with or without BMDCs of normal control mice or OSC-19 tumour-bearing mice were s.c. injected into non-irradiated (non-IR) or pre-IR sites. For the other experiment, OSC-19 cells were injected with or without BM-M1Mφs or BM-M2Mφs.

### Immunofluorescence staining for mouse sample

Tumour samples were fixed by 4% paraformaldehyde cardiac perfusion and then embedded in OCT compound (Sakura Finetek). Frozen sections (5–10 μm) were incubated with the following primary anti-mouse antibodies overnight at 4 °C: CD31 (BD Bioscience), CD11b (BioLegend), Tie-2, F4/80, Gr-1 (eBioscience), VEGFR2 (R&D Systems), CD68 (Serotec), CD206 (Santa Cruz) and iNOS (Abcam). They were then incubated with secondary antibodies for 30 min at room temperature, including Alexa Flour 488, 546, Streptavidin labeled with Alexa Flour 488, 546 and 647 (Thermo Fisher Scientific). Hypoxic areas were detected using pimonidazole hydrochloride staining (Hypoxyprobe^TM^-1 Omni kit, Natural Pharmacia International) according to the manufacturer’s instructions. All sections were mounted in ProLong® Gold Antifade Mountant with DAPI (Thermo Fisher Scientific) and imaged with Leica DMI 4000B fluorescent microscopy (Leica Microsystems Inc.). Isotype-matched IgG was used as a negative control.

### Immunohistochemistry for human samples

Human OSCC specimens of primary tumours and matched recurrences after chemoradiotherapy were obtained from the same individuals by biopsy or surgical resection at the Department of Oral and Maxillofacial Surgery in Yokohama City University Hospital after obtaining informed consent from all subjects. The protocol to use tissue samples for this study was approved by Yokohama City University School of Medicine in accordance with the principles of the Declaration of Helsinki. Paraffin-embedded sections were incubated with anti-human CD11b, CD31, CD206 (Abcam) and CD68 (Dako) overnight at 4 °C. The reaction was visualized with the Vector Stain ABC kit and ImPACT DAB Substrate (Vector Laboratories). Sections were counterstained with haematoxylin and viewed with an OLYMPUS BH2 microscope (Olympus).

### Matrigel Plug assay

Matrigel (growth factor reduced phenol red-free, Corning; 450 μl) supplemented with 75 ng of basic fibroblast growth factor (bFGF) (Peprotech) was mixed with BM-M2Mφs (1 × 10^6^ cells) and injected into the flank of female BALB/c nude mice. Matrigel with bFGF served as a control. One week after inoculation, Matrigel plugs were surgically removed, and frozen sections were analysed by immunofluorescence staining for the presence of CD31 endothelial cells.

### Quantification of expression

Quantitative analyses for CD11b, Tie-2, F4/80, Gr-1, VEGFR2, CD68, CD206, iNOS and CD31 were performed by determining the number of positive cells. The signal density of CD31 and pimonidazole were measured using Image J. All analyses were performed in at least three randomly photographed fields using the ×20 or ×40 objectives and ×10 eyepieces of the fluorescence microscope. Three to eight animals per group were used; the error bars shown in the figures indicate SD or SEM.

### Flow cytometric analysis

Mouse whole blood was collected with 1.5% EDTA-2Na used as the anticoagulation agent, and red blood cells were eliminated by a lysing buffer (BD Pharmingen). Leukocyte cells and BMDCs of normal control mice or OSC-19 tumour-bearing mice were stained with CD11b-PE antibody (BD Pharmingen). For IL-13Rα2 and CD11b double staining, OSC-19 tumours grown at non-IR sites or pre-IR sites in nude mice were minced and enzymatically digested with 3 mg/ml Type VI collagenase and 2 U/ml hyaluronidase (SIGMA). They were further treated with 0.1 mg/ml DNase I (Wako) and 0.09% NH_4_Cl and strained through a 40 μm cell strainer. Single cells were incubated with IL-13Rα2-biotin (R&D systems) and CD11b-PE antibodies (BD Pharmingen), followed by Streptavidin-APC antibody (eBioscience). All samples were pre-incubated with Fc-block (BioLegend) prior to staining. Analysis was performed using MoFlo® Astrios (Beckman Coulter) and Summit Software ver. 4.3 (Beckman Coulter).

### Statistical Analysis

Statistical analyses for multiple-group comparison were analysed by one-way ANOVA. For two-group comparisons, Student’s *t*-test was used (GraphPad Prism software version 5.02). *P* values (exact significance) less than 0.05 were considered statistically significant.

## Additional Information

**How to cite this article**: Okubo, M. *et al.* M2-polarized macrophages contribute to neovasculogenesis, leading to relapse of oral cancer following radiation. *Sci. Rep.* **6**, 27548; doi: 10.1038/srep27548 (2016).

## Supplementary Material

Supplementary Information
