# Supplementary material for: M2-polarized macrophages contribute to neovasculogenesis, leading to relapse of oral cancer following radiation
Source: Sci Rep. 2016 Jun 8;6:27548. doi: 10.1038/srep27548 (PMC4897643; doi:10.1038/srep27548)
Supplement: Supplementary Information [file srep27548-s1.pdf]

Supplementary information

M2-polarized macrophage contributes the neovasculogenesis, leading relapse of oral cancer following radiation

Makiko Okubo[1], Mitomu Kioi[1*], Hideyuki Nakashima[1], Kei Sugiura[1], Kenji Mitsudo[1], Ichiro Aoki[2], Hideki Taniguchi[3] & Iwai Tohnai[1].

[1]Department of Oral and Maxillofacial Surgery, Yokohama City University Graduate School of Medicine, Yokohama, Kanagawa 236-0004, Japan.

[2]Department of Pathology, Yokohama City University Graduate School of Medicine, Yokohama, Kanagawa 236-0004, Japan.

[3]Department of Regenerative Medicine, Yokohama City University Graduate School of Medicine, Yokohama, Kanagawa 236-0004, Japan.

[*]E-mail: kioi@yokohama-cu.ac.jp

Context of supplementary information

1. Supplementary Figures 1-5
2. Supplementary Table 1

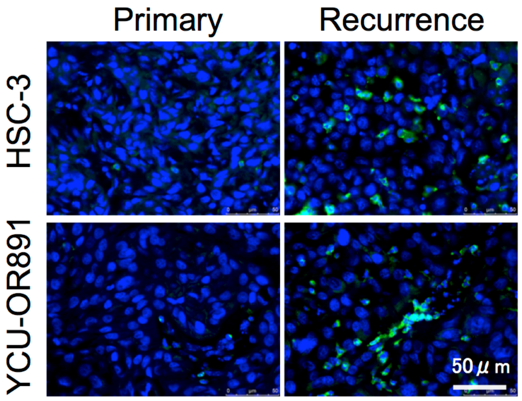

Supplementary Figure S1.

The representative images of IHC for CD11b$^+$ myeloid cells in OSCC tumours before and after irradiation. HSC-3 cells (1×10^7 cells) and YCU-OR891 cells (3×10^6 cells) were s.c. implanted on the back of female BALB/c nude mice, and grown tumours were irradiated in a manner similar to the OSC-19 model. Tumour samples were stained with CD11b antibody.

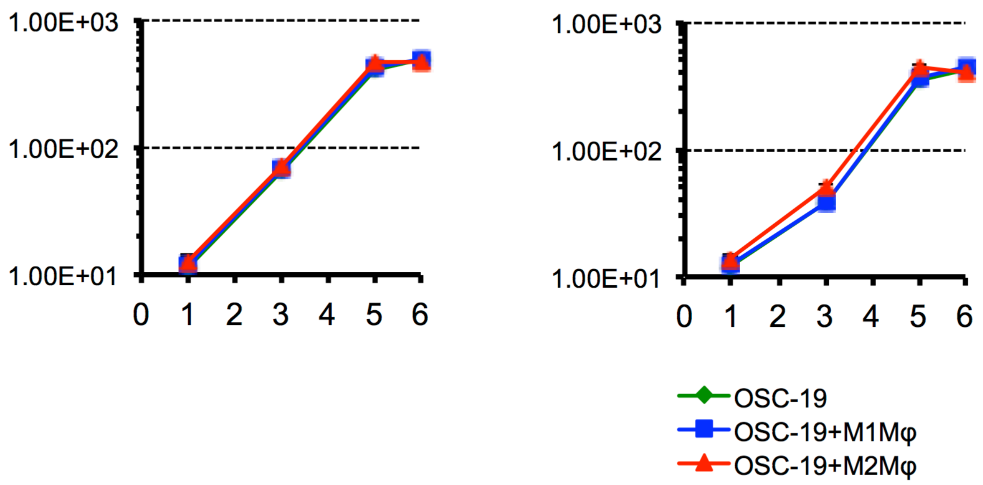

Supplementary Figure S2.

Co-culture experiment of OSC-19-luc cells and BM-M1Mφs or BM-M2Mφs.

OSC-19-luc cells (6×10^3 cells) were co-cultured with BM-M1Mφs or BM-M2Mφs (1.5 or 6×10^3 cells) in 24 well plates in quadruplicate. Bioluminescent signals were determined using the IVIS system on indicated date. Data shown are means ± SD.

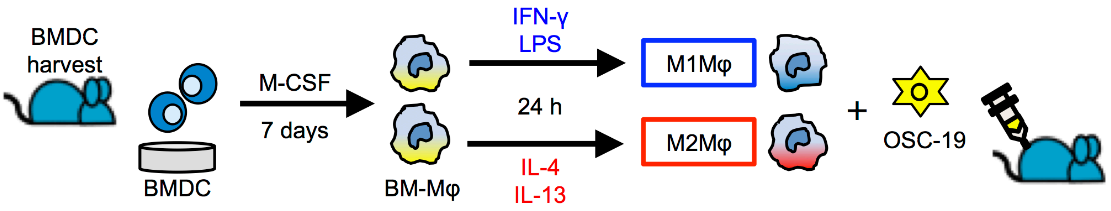

Supplementary Figure S3.

Pattern diagram of the experimental design for co-injection of OSC-19 cells and BM-M1Mφs or BM-M2Mφs. OSC-19 cells were injected into non-IR or pre-IR sites alone or in the presence of BM-M1Mφs or BM-M2Mφs previously prepared *in vitro*.

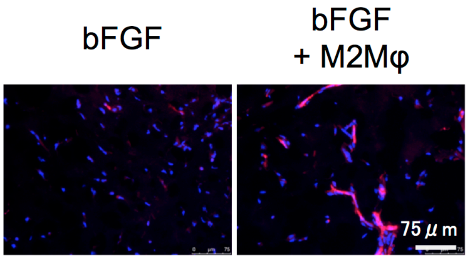

Supplementary Figure S4.

Representative image of Matrigel Plug assay. BM-M2Mφs promoted tube-like formation of $CD31^+$ endothelial cells (red) in Matrigel plug.

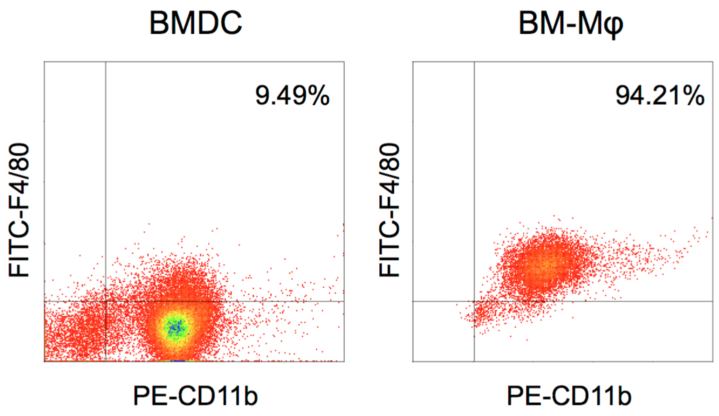

Supplementary Figure S5.

FACS analysis of BMDCs and BM-Mφs. FACS analysis showed that $CD11b^+F4/80^-$ BMDCs harvested from BALB/c nude mice differentiated into $CD11b^+F4/80^+$ BM-Mφs by extended culture with M-CSF for 7 days. BMDC, bone marrow-derived cell; BM-Mφs, bone marrow-derived macrophages; M-CSF, macrophage colony-stimulating factor.

	Forward 5` ⇒ 3`	Reverse 5` ⇒ 3`	Product length
IL-6	GACAAAGCCAGAGTCCTTCAGAGAG	CTAGGTTTGCCGAGTAGATCTC	228(bp)
iNOS	TTTGCTTCCATGCTAATGCGAAAG	GCTCTGTTGAGGTCTAAAGGCTCCG	600
Arg Ⅰ	CAGAAGAATGGAAGAGTCAG	CAGATATGCAGGGAGTCACC	249
CD206	GCAAATGGAGCCGTCTGTGC	CTCGTGGATCTCCGTGACAC	300
MMR	AGCTACCATGGCATGAAGCAGAGA	ACCCATTCGAAGGCATTCCAGAGA	464
IL-13Rα2	CGCATTTGTCAGAGCATTGT	CCAAGCCCTCATACCAGAAA	216
GAPDH	TATGTCGTGGAGTCTACTGGT	GAGTTGTCATATTTCTCGT	149

Supplementary Table S1.

Primer sequences specification for RT-PCR.
